# Emergence of Huntington Disease in a Man With a Premorbid Criminal Lifestyle

**DOI:** 10.3389/fpsyt.2019.00477

**Published:** 2019-07-02

**Authors:** Jonatan Hedlund, Thomas Masterman

**Affiliations:** ^1^Department for Forensic Psychiatry, National Board of Forensic Medicine, Stockholm, Sweden; ^2^Department of Clinical Neuroscience, Karolinska Institutet, Stockholm, Sweden

**Keywords:** Huntington disease, forensic psychiatry, violence, psychosis, dementia

## Abstract

We present here a case in which Huntington disease (HD) was diagnosed upon forensic-psychiatric evaluation of a 34-year-old male repeat offender. Despite a family history of HD, as well as overt delusions and motor pathology, the disease had not been recognized at an earlier stage, and the patient was serving a prison sentence at the time of diagnosis. The case highlights difficulties court officials may face with regard to identifying severe psychiatric and neurological disorders in repeat offenders. Such offenders’ gradually deteriorating status could be overlooked by the court, even in cases in which a tailored judicial process is warranted. Also, the present case highlights the risk of using antipsychotic medication to treat HD, since it may worsen sufferers’ capacity to recognize emotions in others, thereby increasing the risk of altercations and criminal activity.

## Background

Huntington disease (HD) is an autosomal-dominant, neurodegenerative disorder with a progressive course that typically entails motor and cognitive decline and diverse psychiatric disturbances (1). Its pathogenic mechanisms are, at present, not fully understood, although a faultily encoded version of the protein huntingtin—resulting from a cytosine-adenine-guanine (CAG) trinucleotide expansion in the *HTT* gene—has been shown to cause intracellular toxicity in neural tissue by means of various routes (2). HD usually presents in mid-life and eventually leads to devastating consequences for patients (e.g., inability to work and socially deviant behavior)—necessitating the burdensome involvement of familial caregivers—as well as concerns about developing the disease in relatives at risk (1, 2). Occasionally, as is the case in many other dementing disorders, HD gives rise to criminal behavior (3); although such behavior is sometimes violent in nature, severe crimes, such as homicide or rape, are rarely seen (4). Interestingly, in a recent retrospective medical-record review of 2,397 patients with dementia, criminal behavior was a presenting sign in 5 of 30 patients with HD (3). In addition, retrospective reviews of the life histories of HD patients sometimes uncover evidence of antisocial behavior preceding neurological manifestations of the disease; at the same time, it has been speculated that such behavior may be a psychosocial consequence of growing up in a family in which many relatives suffer from HD (5).

In Sweden, dementing disorders are uncommonly diagnosed during forensic-psychiatric evaluations: Of 1,471 individuals who had undergone evaluations during the years 2008–2010, only 21 were found to fulfill criteria for dementia (none of whom was suffering from HD) (6). Moreover, during the years 1999–2014, only 11 individuals with HD were subjected to forensic-psychiatric evaluations in Sweden, according to data extracted from a nationwide database maintained by the governmental authority the National Board of Forensic Medicine.

Approximately 100,000 criminal offenses are committed in Sweden each year; however, only about 500 offenders undergo forensic-psychiatric evaluation, a mere half of whom are adjudged to have committed their offenses under the influence of a severe mental disorder. Thus, quotidian antisocial mechanisms are far more common causes of crime than the effects of severe mental illness. Against this backdrop, a risk exists that the behavior incurred by gradually evolving dementing disorders—including HD—might, in a repeat offender, be mistaken for habitual unruliness. In this paper, we present a case of HD that was diagnosed during a forensic-psychiatric evaluation. We aim to shed light on HD patients in a criminal context and also highlight some shortcomings in the present criminal justice system. In addition, treatment options with potentially criminogenic effects are briefly discussed.

## Case Presentation

### Case History

A 34-year-old male second-generation Turkish immigrant was sent for a court-ordered forensic-psychiatric evaluation following allegations of coercion against a former girlfriend. Specifically, he had demanded 1.2 million Swedish kronor (equivalent to about €115,000) and two mobile phones; if his demands were not met, he threatened to destroy the girlfriend’s and her family members’ vehicles. Prior to the index offense, since the age of 15 (the age of criminal responsibility in Sweden), the client had repeatedly engaged in criminal activity, resulting in a total of four previous prison sentences and numerous non-custodial sentences. By the time of the evaluation, the client had 25 entries to his name in the Swedish crime registry, including verdicts for rape, assault, and drug offenses. There had been no previous psychiatric contacts, although the client appeared to use cannabis on a daily basis, indicative of a severe addiction problem. The client believed himself to be in good physical condition with the exception of a hereditary, progressive and paternally transmitted bilateral hearing impairment, for which he used hearing devices. His schooling had been problematic owing to learning disabilities, truancy, drug abuse, poor parental support, and criminal activities. At the time of the index offense, the client was single and unemployed and resided with his father in a small apartment. During the course of the forensic-psychiatric evaluation, it was revealed that the client’s brother suffered from HD, and that his mother most likely had suffered from the same disease prior to her death a few years earlier.

### Clinical Presentation

The client was admitted to a ward at the forensic-psychiatric evaluation unit, where he was observed for several weeks and subjected to diagnostic interviews, as well as psychological assessments and laboratory investigations. Gross neurological examination revealed no evident motor or sensory deficits, although moderate facial and bilateral limb dystonia was identified. Choreatic movements, disguised as purposive gestures, were seen in the upper limbs. Further, there was a moderate slowing of saccade initiation and velocity, both horizontally and vertically. Subsequent assessment of his admission status from case notes revealed a total score of 27 points on the *Unified Huntington’s Disease Rating Scale*, which assesses four domains of performance and capacity in HD: motor and cognitive function, behavioral abnormalities, and functional capacity (7). A cognitive impairment became evident upon psychometric testing, as well as during interviews, with results from the former pointing toward the mental capacity of a 7-year-old. In addition, his speech was staccato, and his description of being involved in a “game” in which the plaintiff, a policeman and employees at the forensic-psychiatric evaluation unit were also participating was adjudged to be a delusion. Further, his affect was generally blunted yet characterized by an easily evoked hostility.

In interviews, the client—who upon arrest for the index offense began, while in custody at the unit, serving a prison sentence in accordance with a previous verdict—insisted that his release was imminent, suggesting that he did not fully grasp his legal situation. Information gathered from authorities and relatives described the onset of neurological signs 3 years prior to the index offense, followed by a gradual worsening of the client’s state. Genetic analysis, performed on account of clinical signs of a movement disorder, disclosed a pathological 48-repeat cytosine-adenine-guanine (CAG) expansion in one of the alleles of the *HTT* gene, confirming a diagnosis of HD. Computerized tomography of the brain showed global atrophy of the cortex but no apparent pathology in the caudate nucleus or putamen, regions commonly affected in HD.

## Discussion

Research has shown that the clinical presentation of dementing disorders surprisingly often includes criminal behavior (3). The present case, however, highlights a temporally reversed course: the emergence of a debilitating neurological disease in a person with a premorbid criminal lifestyle. This situation poses a special challenge for court officials in terms of identifying defendants who may be unfit to stand trial or in need of a tailored judicial process. The client in the present case was found to be suffering from a severe mental disorder, a medicolegal term implying the aptness of a sentence of forensic-psychiatric care rather than imprisonment. Yet, at the time of the evaluation, he had already commenced serving a short prison sentence; and, in the preceding years, he had served several additional sentences during a period of time when he was likely experiencing severe psychiatric and cognitive symptoms. Possibly, identification of the client’s incipient neurological disorder was made difficult by his comorbid hearing impairment. At the same time, another plausible—perhaps more troublesome—explanation is that the court simply regarded him as a hopeless recidivist and may thus have remained incognizant of his cognitive decline and delusional behavior.

Prior research has highlighted the fact that individuals suffering from HD often have difficulties recognizing negative affects (expressed either vocally or facially), such as anger and disgust (8). The present case involved multiple instances of unwelcome contact on the part of our client, and the court had several times issued restraining orders vis-à-vis the plaintiff. It is worth noting that the client’s criminal record for the period preceding HD onset (3 years prior to the index crime) contains only one entry regarding violation of a restraining order. A phenomenon related to such violations—stalking—is described by Soliman and co-workers (9) in a woman whose unwanted surveillance of her therapist was posited to be caused by an early manifestation of basal-ganglia dysfunction in HD. Indeed, the reduced ability in sufferers of HD to recognize emotions may very well have been a contributing criminogenic factor in the present case; also, recent research has shown that HD is associated with visual-processing deficits—a phenomenon that could further compound the effects of poor emotion recognition (10). In addition, research has shown empathy deficits in HD patients, a finding consistent with an increased risk of criminality (11). Furthermore, it has been reported that antipsychotic medication—which is commonly used to treat aggressive behavior in HD patients (12)—worsened emotion recognition in an HD patient sample, whereas selective serotonin-reuptake inhibitors had the opposite effect (13). Thus, in order to prevent interpersonal violence on the part of HD patients, it is arguably advisable to reassess medication alternatives; indeed, treating the highly variable symptomatology of HD is a complex task, as described in recent expert-based treatment guidelines (14).

## Concluding Remarks

Psychometrically identifiable features in HD appear to be important in the context of analyzing circumstances occasioning criminal acts, as well as in the context of considering medication alternatives. Moreover, it is imperative that officials within the judicial system monitor repeat offenders for the emergence of abnormal signs, in order to avoid imprisonment of severely mentally disabled individuals. Finally, it would be beneficial if healthcare personnel at correctional facilities and remand prisons routinely investigated family history in order to uncover rare heritable diseases that may call for a tailored judicial process. In fact, recent research suggests that even common, acquired neurological disorders—not least encephalopathy resulting from repeated concussions (15)—can insidiously give rise to socially inappropriate behavior, including crime.

## Ethics Statement

Written informed consent was obtained from the client for publication of this case report. A copy of the written consent is available for review from the first author.

## Author Contributions

Both authors (TM and JH) have participated in reviewing medical records and drafting the manuscript.

## Conflict of Interest Statement

The authors declare that the research was conducted in the absence of any commercial or financial relationships that could be construed as a potential conflict of interest.
